# English teachers’ social-emotional competence and occupational burnout: the mediating role of teaching efficacy and the moderating role of perceived organizational support

**DOI:** 10.3389/fpsyg.2026.1972636

**Published:** 2026-09-22

**Authors:** Yanyan Zhang

**Affiliations:** School of Foreign Languages, Xinyang University, Xinyang, China

**Keywords:** English teachers, job demands-resources model, moderated mediation, occupational burnout, perceived organizational support, self-efficacy, social-emotional competence, teaching efficacy

## Abstract

Occupational burnout among English teachers has become a pressing concern, yet the mechanisms linking teachers’ social-emotional competence (SEC) to burnout remain incompletely understood. This study examined a moderated mediation model in which teaching efficacy mediates the association between SEC and burnout, and perceived organizational support (POS) moderates the SEC-to-teaching efficacy pathway. A cross-sectional survey was administered to 412 English teachers from 24 primary and secondary schools across three cities in Henan Province, China, using measures of SEC, teaching efficacy, POS, and burnout. Confirmatory factor analysis supported the hypothesized four-factor measurement model, and convergent and discriminant validity were established through average variance extracted, composite reliability, and the Fornell-Larcker criterion. Moderated mediation analysis revealed that SEC was negatively associated with burnout, that this association was partially mediated by teaching efficacy, and that POS moderated the SEC-to-teaching efficacy pathway, such that the association was stronger among teachers who perceived higher organizational support. The conditional indirect effect of SEC on burnout through teaching efficacy was stronger at higher levels of POS, and Johnson-Neyman analysis indicated that this moderated pathway held for the overwhelming majority of the sample. These findings suggest that both personal competencies and organizational context shape English teachers’ occupational well-being, and they are discussed with respect to theory, the Chinese cultural context, and implications for teacher development and school management. The findings can provide a reference for practice in basic education and also offer implications for talent cultivation in college-level English teacher education programs.

## 1 Introduction

### 1.1 Teacher burnout as an occupational challenge

Occupational burnout, broadly defined as a psychological syndrome characterized by emotional exhaustion, depersonalization, and reduced personal accomplishment (Maslach et al., 2001), represents a pervasive challenge in the teaching profession. Teachers are particularly vulnerable to burnout due to the emotionally demanding nature of their work, frequent exposure to interpersonal stressors, and high-stakes accountability pressures; meta-analytic evidence indicates that emotional labor is reliably associated with burnout across occupational groups, with emotional intelligence serving as a protective factor (Chen et al., 2024). Burnout among teachers has been linked to reduced teaching effectiveness, higher turnover intentions, and detrimental student outcomes, with teacher-, student-, and organization-level variables each contributing to its explanation (Saloviita and Pakarinen, 2021). In China, studies have documented substantial burnout among teachers, particularly in rural and under-resourced settings (Zhao et al., 2023), and reviews specific to English teachers report pervasive workload and emotional demands in this group (Zheng, 2022), underscoring the urgency of identifying protective factors.

### 1.2 Teachers’ social-emotional competence as a personal resource

social-emotional competence (SEC) has emerged as a promising personal resource that may protect teachers against burnout. SEC encompasses the capacities to recognize and manage emotions, develop caring and concern for others, establish positive relationships, make responsible decisions, and handle challenging situations constructively (Jennings and Greenberg, 2009). Within the prosocial classroom model, teachers’ SEC and wellbeing are foundational to healthy teacher-student relationships, effective classroom management, and successful social-emotional learning program implementation (Jennings and Greenberg, 2009; Fabris et al., 2022). Empirically, recent meta-analytic evidence indicates that emotional intelligence, a construct closely related to SEC, is negatively associated with burnout among foreign language teachers specifically (Wang and Wang, 2022), and longitudinal work has identified teachers’ perceived SEC as a meaningful predictor of occupational outcomes (Collie, 2025). Beyond the classroom, teachers increasingly function as relational anchors for their students: contemporary research conceptualizes teachers as important non-parental attachment figures whose own attachment orientations shape the quality of their relationships with students (Nulman and Alkalay, 2023), and the quality of teacher-student relationships reliably tracks student adjustment (Fabris et al., 2022; Wang and Xian, 2024). Teachers are also routinely called upon to recognize and respond to students who have experienced adversity or trauma, a role that itself imposes emotional demands on teachers of English as an additional language and other subject teachers alike (Fabris et al., 2024; Kostouros et al., 2023). These relational and trauma-responsive responsibilities make SEC not merely an instructional asset but an occupational requirement, and teachers with limited SEC may be exposed to heightened emotional strain as a result (Poulou and Garner, 2024). Teachers who possess higher SEC are better equipped to regulate their emotions, build supportive relationships, and navigate interpersonal challenges, all of which may reduce vulnerability to burnout (Zhang et al., 2023; Ma and Niu, 2025). Accordingly, we hypothesized that: (H1) SEC is significantly and negatively associated with burnout.

### 1.3 Teaching efficacy as a mediating mechanism

Teaching efficacy, defined as a teacher’s belief in their capability to organize and execute courses of action required to successfully accomplish a specific teaching task (Tschannen-Moran and Woolfolk Hoy, 2001), represents a potential mechanism linking SEC to burnout. Self-efficacy theory (Bandura, 1997) posits that mastery experiences, that is, successes in managing challenging situations, are the most powerful source of efficacy beliefs. Teachers with higher SEC are more likely to create emotionally supportive classroom environments, handle disruptive behavior effectively, and maintain positive relationships with students and colleagues, thereby accumulating mastery experiences that build teaching efficacy (Nulman and Alkalay, 2023; Poulou and Garner, 2024), a pathway consistent with evidence that teacher emotional intelligence is positively associated with self-efficacy and work engagement (Wang, 2022). In turn, teaching efficacy operates as a personal resource that buffers against burnout by promoting a sense of professional competence and reducing the appraisal of teaching demands as overwhelming (Skaalvik and Skaalvik, 2010; Kim and Burić, 2020; Li, 2023; Hlado et al., 2025). Accordingly, we hypothesized that: (H2) teaching efficacy mediates the negative association between SEC and burnout.

### 1.4 The moderating role of perceived organizational support

While the SEC-teaching efficacy link is theoretically well-grounded, its magnitude is likely to depend on contextual factors. Perceived organizational support (POS), defined as employees’ global perception that their organization values their contributions and cares about their wellbeing (Eisenberger et al., 1986), represents a key contextual resource that may shape the extent to which SEC translates into professional competence. The Job Demands-Resources (JD-R) model (Bakker and Demerouti, 2007) posits that personal resources (e.g., SEC) and job resources (e.g., POS) interact synergistically in predicting employee wellbeing. We therefore hypothesized that POS would moderate the SEC-to-teaching efficacy pathway (the a-path) specifically, rather than the teaching efficacy-to-burnout pathway (the b-path). This specification is grounded in two considerations. First, from a theoretical standpoint, organizational support is most proximally relevant to the process by which personal competencies are expressed, recognized, and consolidated into professional beliefs: supportive environments provide the autonomy, feedback, and resources that enable teachers to translate their SEC into concrete teaching behaviors and mastery experiences (Kurtessis et al., 2017). Second, from an empirical standpoint, research on perceived organizational support indicates that its effects are strongest where personal resources meet enabling conditions (Kurtessis et al., 2017), and in the Chinese context specifically, POS has been shown to shape teacher motivation, professional beliefs, and work engagement (Chen, 2025; Mei and Alias, 2026). Nevertheless, we acknowledge that the choice of Model 7 represents a theoretically informed but not exhaustive specification, and alternative moderation models are examined as sensitivity analyses in the present study. Accordingly, we hypothesized that: (H3) POS moderates the SEC-to-teaching efficacy pathway, such that the association is stronger at higher POS.

### 1.5 The present study

The present study examines a moderated mediation model in which teaching efficacy mediates the SEC-burnout relationship, and POS moderates the SEC-to-efficacy pathway (Figure 1). English teachers occupy a distinctive niche within the educational ecosystem that may render the SEC-burnout nexus especially salient. English language teaching in China involves high levels of emotional labor: teachers must maintain an enthusiastic, encouraging demeanor to lower students’ foreign language anxiety (Horwitz et al., 1986) while simultaneously managing the pressure of high-stakes examinations (Zheng, 2022), and novice English teachers in rural schools must actively navigate emotional challenges in their daily work (Yao et al., 2023). Moreover, the emotional labor profiles of Chinese teachers have been shown to relate systematically to job demands, resources, and burnout (Hu et al., 2023). SEC is particularly important in this context because effective language instruction depends on the teacher’s ability to create a psychologically safe, emotionally engaging classroom environment.

**Figure 1:**
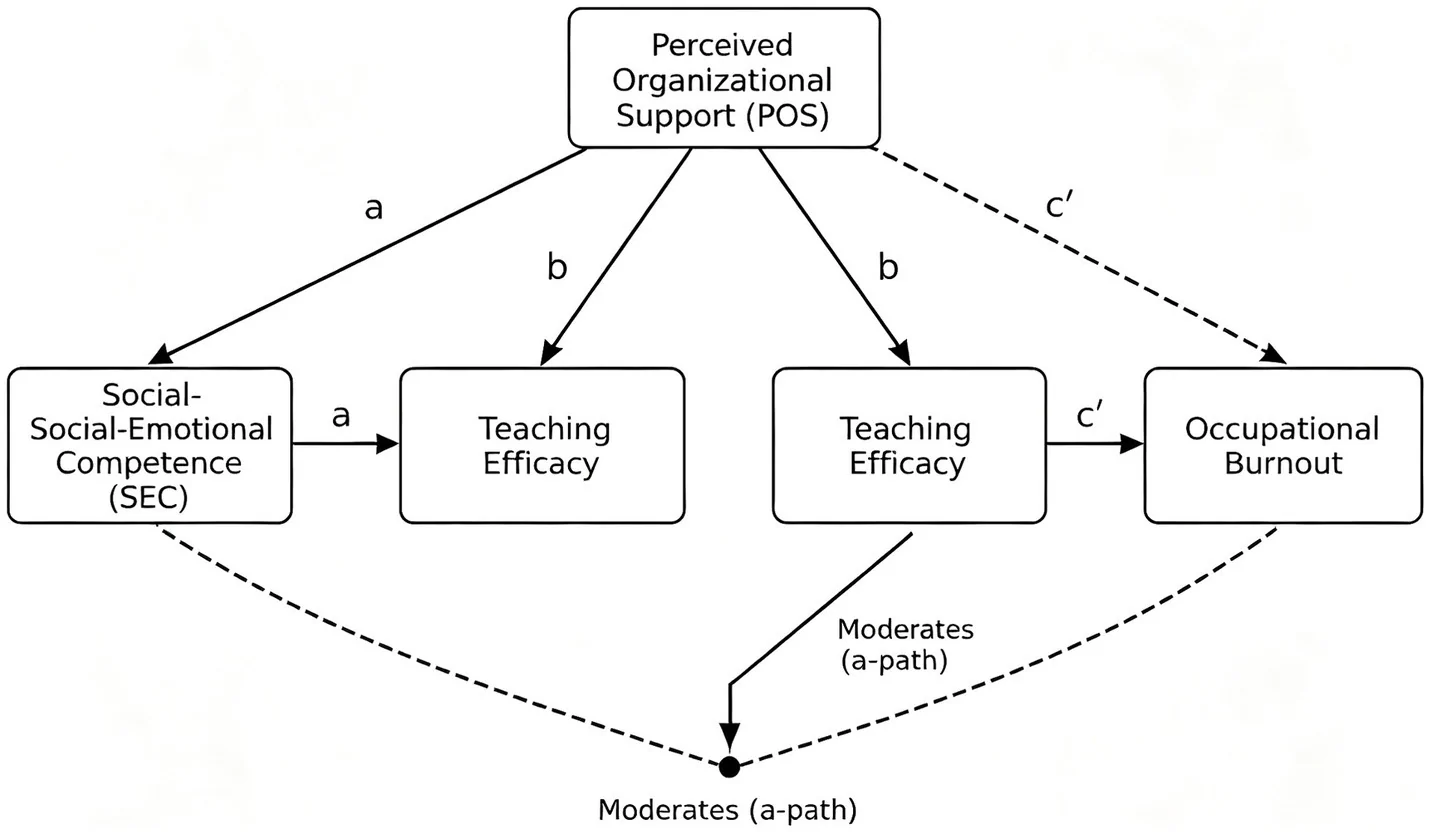
Conceptual moderated mediation model. Social-emotional competence (SEC) is hypothesized to influence occupational burnout both directly and indirectly through teaching efficacy. Perceived organizational support (POS) moderates the SEC-to-teaching efficacy pathway (a-path). Covariates (gender, age, teaching experience, school level) are included but not depicted for clarity.

This study extends prior work in several ways. Zhang et al. (2025) examined how SEC relates to job burnout among Chinese rural kindergarten teachers, finding that organizational trust moderated the SEC-burnout association through a chain mediation involving self-efficacy and job satisfaction. The present study differs from Zhang et al. (2025) in four respects: (a) population, that is, English teachers in primary and secondary schools, who face distinctive emotional labor demands including foreign language anxiety management, versus rural kindergarten teachers; (b) moderator, that is, perceived organizational support (POS), a specific job resource within the JD-R framework, versus organizational trust as a broader climate variable; (c) mediator, that is, teaching efficacy as a single mediator, versus a chain mediation through self-efficacy and job satisfaction; and (d) analytical approach, that is, PROCESS Model 7 with Johnson-Neyman analysis to identify the precise transition point of moderation, versus standard moderation analysis. The cultural context of the study also warrants emphasis. In Chinese schools, hierarchical organizational structures, collectivist norms, and high parental expectations for English achievement shape both the expression of teacher SEC and the meaning of organizational support; cross-cultural research has documented systematic differences in teacher-student relationship quality between Chinese and Western settings (Fabris et al., 2023), suggesting that mechanisms identified in Western samples cannot be assumed to generalize. Building on the rationale developed above and on the hypothesized mediation and moderation pathways, we finally hypothesized that: (H4) the indirect effect of SEC on burnout through teaching efficacy is conditional on POS, being stronger at higher POS.

## 2 Materials and methods

### 2.1 Participants and procedure

This study employed a cross-sectional survey design. The target population was English teachers working in primary and secondary schools in Henan Province, China. *A priori* power analysis using G*Power 3.1 indicated that a minimum sample of 199 participants would be required to detect a medium effect size (*f*^2^ = 0.15) in multiple regression with seven predictors at *α* = 0.05 and power = 0.95. To account for potential non-response and to enable subgroup analyses, we oversampled.

A total of 460 questionnaires were distributed and collected through a combination of convenience and snowball sampling across 24 schools in three cities (Zhengzhou, Luoyang, and Xinyang), with initial contacts established through school administrators in eight schools per city; each participating teacher was subsequently invited to forward the survey link to two to three colleagues, constituting the snowball component. Data collection took place between March and May 2025. Questionnaires were administered in two formats: paper-and-pencil questionnaires completed during faculty meetings and an online survey platform (Wenjuanxing). After excluding 48 responses due to incomplete data (*n* = 31) or failure on attention-check items (*n* = 17), the final valid sample comprised 412 teachers (paper-and-pencil mode, *n* = 185; online mode, *n* = 227), corresponding to an effective response rate of 89.6%. The number of valid respondents per school ranged from 9 to 31 (*M* = 17.2, SD = 5.4). This mixed-mode design was adopted for practical feasibility, as paper-and-pencil and online administration of the same instrument has been shown to yield comparable data (Bryan et al., 2022; Heimel et al., 2024). Preliminary comparisons indicated that the two administration modes did not differ significantly in demographic characteristics or any study variable (see Results), supporting the pooling of the two subsamples.

The demographic characteristics of the sample are presented in Table 1. Of the 412 participants, 301 (73.1%) were female and 111 (26.9%) were male. The mean age was 36.5 years (SD = 8.2, range = 23–58), and the mean teaching experience was 11.3 years (SD = 7.4, range = 1–35). In terms of school level, 186 (45.1%) taught in primary schools, 190 (46.1%) in junior secondary schools, and 36 (8.7%) in senior secondary schools. The majority held a bachelor’s degree (67.5%), with 31.3% holding a master’s degree and 1.2% holding a doctoral degree. Regarding professional title, 145 (35.2%) held junior titles, 201 (48.8%) intermediate titles, and 66 (16.0%) senior titles. The mean weekly teaching hours were 16.8 (SD = 4.3, range = 8–28). Most participants worked in public schools (*n* = 358, 86.9%), with 54 (13.1%) from private schools.

**Table 1:** Demographic characteristics of the sample (*N* = 412).

Characteristic	Category	*n*	%
Gender	Female	301	73.1
	Male	111	26.9
Age (years)	M (SD) = 36.5 (8.2), range = 23–58		
Teaching experience (years)	M (SD) = 11.3 (7.4), range = 1–35		
School level	Primary	186	45.1
	Junior secondary	190	46.1
	Senior secondary	36	8.7
Education	Bachelor’s	278	67.5
	Master’s	129	31.3
	Doctoral	5	1.2
Professional title	Junior	145	35.2
	Intermediate	201	48.8
	Senior	66	16.0
Weekly teaching hours	M (SD) = 16.8 (4.3), range = 8–28		
School type	Public	358	86.9
	Private	54	13.1
City	Zhengzhou	168	40.8
	Luoyang	142	34.5
	Xinyang	102	24.8

*N* = 412. Percentages may not sum to 100 due to rounding.

The study was approved by the ethics review board of Xinyang University (approval no. XYU-2025-EDU-031) prior to data collection. All participants provided informed consent in accordance with the approved procedure (written consent for the paper-and-pencil mode; electronic consent for the online mode) prior to participation. Participation was voluntary, and respondents were assured of anonymity and confidentiality.

### 2.2 Measures

social-emotional competence was assessed using the Teacher Social-Emotional Competence Scale (TSEC-S), a 25-item self-report measure adapted from the five-competency framework of the (Collaborative for Academic, Social, and Emotional Learning [CASEL], 2020) and the prosocial classroom model (Jennings and Greenberg, 2009). The scale captures five dimensions—self-awareness (5 items), self-management (5 items), social awareness (5 items), relationship skills (5 items), and responsible decision-making (5 items)—with content adapted to the classroom context. An example item is, “I can identify my emotions accurately during classroom interactions.” The instrument was developed through a systematic scale construction procedure. First, an initial pool of 30 items was generated based on CASEL’s competency definitions and reviewed by five experts in educational psychology (three specializing in social-emotional learning and two in educational measurement). The content validity index (CVI) was computed for each item; items with item-level CVI (I-CVI) below 0.80 were revised or deleted, yielding a scale-level CVI (S-CVI) of 0.89. Based on expert feedback and item-level CVI, five items were removed, resulting in the final 25-item scale. A pilot study was conducted with 50 English teachers (not included in the main sample) to evaluate preliminary psychometric properties. The pilot sample demonstrated acceptable internal consistency (Cronbach’s *α* = 0.89). Convergent validity was assessed by correlating TSEC-S scores with the Trait Emotional Intelligence Questionnaire Short Form (TEIQue-SF; Petrides, 2009), yielding a moderate positive correlation (*r* = 0.52, *p* < 0.01), supporting the convergence of TSEC-S with an established emotional competence measure. Test–retest reliability over a 4-week interval was *r* = 0.78. All items were rated on a 5-point Likert scale (1 = strongly disagree to 5 = strongly agree). In the main study, the Cronbach’s alpha for the total scale was 0.91, with subscale alphas ranging from 0.78 to 0.86. Confirmatory factor analysis in the present sample supported the five-factor structure (*χ*^2^/df = 2.41, CFI = 0.93, RMSEA = 0.059). The complete item pool, expert review documentation, item-level content validity indices, pilot results, and the five-factor CFA of the TSEC-S in the present sample are provided in the Supplementary material.

Teaching efficacy was measured using the 12-item short form of the Teachers’ Sense of Efficacy Scale (TSES; Tschannen-Moran and Woolfolk Hoy, 2001). This instrument captures three dimensions—instructional strategies, classroom management, and student engagement—with four items each. Participants rated their confidence on a 9-point scale (1 = nothing, 5 = very little, 9 = a great deal). An example item is, “How much can you do to motivate students who show low interest in schoolwork?” Cronbach’s alpha in the present sample was 0.89. The three-factor structure was confirmed by CFA (*χ*^2^/df = 2.93, CFI = 0.92, RMSEA = 0.071).

Perceived organizational support was assessed using the 8-item Short Survey of Perceived Organizational Support (SPOS; Eisenberger et al., 1997). The Chinese version was developed through a standard translation and back-translation procedure (Brislin, 1970) by two bilingual researchers and has been validated in previous Chinese studies (Liu et al., 2013). Teachers indicated their agreement with each item on a 7-point Likert scale (1 = strongly disagree to 7 = strongly agree). A sample item is, “My school values my contributions to its well-being.” The Cronbach’s alpha was 0.87 in this study. CFA results supported the unidimensional structure (*χ*^2^/df = 3.12, CFI = 0.95, RMSEA = 0.073).

Occupational burnout was measured using the 15-item Maslach Burnout Inventory-Educators Survey (MBI-ES; Maslach and Jackson, 1981; Maslach et al., 1996), Chinese version (Li, 2003). The MBI-ES comprises three subscales: emotional exhaustion (5 items), depersonalization (5 items), and personal accomplishment (5 items, reverse-scored). Items are rated on a 7-point frequency scale (0 = never to 6 = every day). A composite burnout score was computed by averaging the three subscale scores, with higher scores indicating greater burnout. The use of a composite score was justified by the moderate-to-strong intercorrelations among the three dimensions in the present sample (ranging from *r* = −0.42 to *r* = 0.51), which supports the presence of a higher-order burnout construct, and by the theoretical conceptualization of burnout as a unified syndrome (Maslach et al., 2001). We acknowledge that this approach is contested by some scholars who advocate dimension-specific analysis; the dimension-level descriptive statistics and correlations are reported in the Results for readers interested in subscale-level patterns. The composite alpha was 0.85, with subscale alphas of 0.88 (emotional exhaustion), 0.79 (depersonalization), and 0.76 (personal accomplishment).

Given the established associations between demographic characteristics and both burnout and teaching efficacy, gender, age, teaching experience, and school level were included as covariates in all subsequent analyses. The correlations between covariates and core study variables are presented in Table 2.

**Table 2:** Descriptive statistics, normality indicators, and zero-order correlations among study variables (*N* = 412).

Variable	M	SD	Skew	Kurt	1	2	3	4
1. SEC	3.72	0.58	−0.21	0.04	–			
2. Teaching efficacy	5.31	1.12	−0.35	0.18	0.44^***^	–		
3. POS	4.85	0.92	−0.48	0.41	0.29^***^	0.31^***^	–	
4. Burnout	2.86	0.81	0.28	−0.15	−0.40^***^	−0.35^***^	−0.26^***^	–
5. Gender	–	–	–	–	−0.07	−0.08	−0.05	0.10^*^
6. Age	36.50	8.20	0.21	−0.42	0.12^**^	0.10^*^	0.06	−0.05
7. Teaching exp.	11.30	7.40	0.58	−0.18	0.10^*^	0.08	0.04	−0.03
8. School level	–	–	–	–	0.02	0.10^*^	0.03	0.06

*N* = 412. SEC, social-emotional competence; POS, perceived organizational support; Teaching exp., teaching experience. Gender coded 0 = male, 1 = female. School level coded 1 = primary, 2 = junior secondary, 3 = senior secondary. Skew = skewness; Kurt = kurtosis. All skewness and kurtosis values are within ±2, indicating acceptable normality. ^*^p < 0.05; ^**^p < 0.01; ^***^p < 0.001.

### 2.3 Data analysis strategy

All analyses were conducted using SPSS 26.0, AMOS 26.0, and the PROCESS macro (Hayes, 2018). Preliminary analyses included descriptive statistics (M, SD, range, skewness, kurtosis), internal consistency reliability estimates (Cronbach’s *α*), and zero-order correlations. Normality was assessed by examining skewness and kurtosis values, with values within ±2 indicating acceptable normality (Field, 2018). Group differences on key variables by gender, school level, and education were examined using independent-samples *t*-tests and one-way ANOVA. McDonald’s omega (*ω*) was computed as a complementary internal consistency estimate, given that alpha assumes essential tau-equivalence (McDonald, 1999; Bell et al., 2023).

Confirmatory factor analysis (CFA) was performed using AMOS 26.0 to evaluate the measurement model before testing the structural model. Model fit was evaluated using *χ*^2^/df (≤3 acceptable), CFI (≥0.90), TLI (≥0.90), RMSEA (≤0.08), and SRMR (≤0.08) (Hu and Bentler, 1999). Convergent validity was assessed through average variance extracted (AVE ≥ 0.50) and composite reliability (CR ≥ 0.70; Fornell and Larcker, 1981). Discriminant validity was evaluated using the Fornell-Larcker criterion, which requires that the square root of AVE for each construct exceeds its correlations with all other constructs. Alternative CFA models (three-factor, two-factor, and one-factor) were compared using *χ*^2^ difference tests and fit indices to establish discriminant validity.

The moderated mediation hypotheses were tested using Hayes’s PROCESS macro (Model 7), which estimates the conditional indirect effect of the independent variable on the dependent variable through a mediator at different levels of a moderator. SEC was specified as the independent variable, teaching efficacy as the mediator, burnout as the dependent variable, and POS as the moderator of the SEC-to-efficacy path. Continuous predictors were mean-centered prior to computing the interaction term. The indirect effect was estimated using 5,000 bootstrap samples to construct 95% bias-corrected confidence intervals; indirect effects were deemed significant when the confidence interval excluded zero. The index of moderated mediation was computed to test whether the indirect effect varied significantly across levels of POS. The Johnson-Neyman technique was applied to identify the precise transition point of POS at which the SEC-teaching efficacy simple slope shifted from non-significant to significant (Preacher et al., 2006). The incremental contribution of the interaction term was assessed using Δ*R*^2^. As a sensitivity analysis, alternative specifications were estimated in which POS moderated the mediator-to-outcome path (Model 14) or the direct SEC-burnout path, to examine whether the moderating role of POS was specific to the SEC-to-teaching efficacy pathway.

Common method bias was assessed using two approaches. First, Harman’s single-factor test was conducted. Second, an unmeasured latent method factor (ULMF) was added to the CFA model, with all items loading on both their substantive factor and the method factor. The change in fit indices (ΔCFI, ΔRMSEA) and the pattern of factor loadings were examined to evaluate the extent of method bias (Podsakoff et al., 2003).

Because the data were collected from 24 schools, we examined the potential clustering effect. The intraclass correlation coefficient (ICC) for burnout at the school level was computed to evaluate the proportion of between-school variance. An ICC below 0.05 is generally considered to indicate negligible clustering effects (Hox, 2010).

## 3 Results

### 3.1 Descriptive statistics, normality, and correlations

Preliminary comparisons between the paper-and-pencil (*n* = 185) and online (*n* = 227) subsamples revealed no significant differences in the distribution of gender, *χ*^2^(1) = 0.62, *p* = 0.431, or school level, *χ*^2^(2) = 1.14, *p* = 0.565, and no significant differences in SEC, *t*(410) = 0.87, *p* = 0.386, teaching efficacy, *t*(410) = 1.02, *p* = 0.309, POS, *t*(410) = 0.74, *p* = 0.459, or burnout, *t*(410) = 0.91, *p* = 0.363. The two subsamples were therefore pooled for all subsequent analyses.

Descriptive statistics, normality indicators, and zero-order correlations among the study variables are presented in Table 2. All variables, including the covariates, showed acceptable normality, with skewness values ranging from −0.48 to 0.58 and kurtosis values ranging from −0.42 to 0.41, all well within the ±2 threshold. SEC was negatively correlated with burnout (*r* = −0.40, *p* < 0.001) and positively correlated with teaching efficacy (*r* = 0.44, *p* < 0.001) and POS (*r* = 0.29, *p* < 0.001). Teaching efficacy was negatively correlated with burnout (*r* = −0.35, *p* < 0.001) and positively correlated with POS (*r* = 0.31, *p* < 0.001). POS was negatively correlated with burnout (*r* = −0.26, *p* < 0.001). The pattern of correlations was consistent with the hypothesized model.

At the sub-dimension level, SEC subscale scores ranged from *M* = 3.65 (self-management) to *M* = 3.80 (social awareness), teaching efficacy subscale scores ranged from *M* = 5.23 (student engagement) to *M* = 5.42 (instructional strategies), and burnout subscale scores ranged from *M* = 2.11 (emotional exhaustion) to *M* = 4.02 (personal accomplishment, reverse-scored), with a depersonalization mean of 2.45. The three burnout subscales showed moderate intercorrelations (emotional exhaustion and depersonalization: *r* = 0.51, *p* < 0.001; emotional exhaustion and personal accomplishment: *r* = −0.42, *p* < 0.001; depersonalization and personal accomplishment: *r* = −0.38, *p* < 0.001), supporting the use of a composite score while also indicating meaningful dimensional variation.

Group differences on key variables were examined to characterize the sample. An independent-samples *t*-test revealed that female teachers reported significantly higher burnout than male teachers (female: *M* = 2.96, SD = 0.83; male: *M* = 2.79, SD = 0.79; *t*(410) = 2.14, *p* = 0.033, *d* = 0.21), but no significant gender differences were found for SEC (*p* = 0.148) or teaching efficacy (*p* = 0.096). One-way ANOVA revealed significant differences in teaching efficacy across school levels (*F*(2, 409) = 4.23, *p* = 0.015), with senior secondary teachers reporting higher efficacy (*M* = 5.55, SD = 1.20) than primary teachers (*M* = 5.18, SD = 1.10). No significant education-level differences were found for SEC (*p* = 0.062).

### 3.2 Common method bias and clustering

Harman’s single-factor test indicated that no single factor accounted for more than 38.4% of the variance, which is below the 50% threshold. The ULMF analysis revealed minimal improvement in model fit (ΔCFI = 0.012, ΔRMSEA = −0.003), and no item loaded more on the method factor than on its substantive factor. Together, these results suggest that common method bias was not a serious threat. It should nevertheless be acknowledged that these procedures cannot entirely rule out consistency motifs, social desirability, or negative affectivity as partial explanations of the observed covariation; this possibility is considered further in the Limitations.

The ICC for burnout at the school level was 0.043, which is below the 0.05 threshold, suggesting that the clustering effect was negligible and that single-level analysis was appropriate. Nevertheless, we acknowledge this as a limitation and discuss it further below.

### 3.3 Measurement model

A confirmatory factor analysis was conducted to evaluate the four-factor measurement model (SEC, teaching efficacy, POS, and burnout as distinct latent factors). The model demonstrated acceptable fit: *χ*^2^(131) = 373.35, *χ*^2^/df = 2.85, CFI = 0.94, TLI = 0.92, RMSEA = 0.067 (90% CI [0.059, 0.075]), and SRMR = 0.05. All factor loadings were significant (*p* < 0.001) and ranged from 0.52 to 0.86.

The five-factor structure of the TSEC-S was further evaluated in the present main sample. The five-factor model demonstrated acceptable fit, *χ*^2^/df = 2.41, CFI = 0.93, TLI = 0.91, RMSEA = 0.059, SRMR = 0.05, with all standardized loadings significant (*p* < 0.001) and ranging from 0.53 to 0.81, and factor correlations between 0.38 and 0.62; the five-factor model fit substantially better than a single-factor model (ΔCFI = 0.21, ΔRMSEA = 0.075). The detailed results, including the factor correlation matrix, are provided in the Supplementary material.

Convergent validity was supported by average variance extracted (AVE) values ranging from 0.48 to 0.54 (all ≥ or approaching the 0.50 threshold) and composite reliability (CR) values ranging from 0.83 to 0.88 (all ≥ 0.70), as shown in Table 3. Discriminant validity was established through the Fornell-Larcker criterion: the square root of AVE for each construct (√AVE = 0.69–0.73) exceeded the construct’s correlations with all other constructs (maximum *r* = 0.44).

**Table 3:** Reliability, convergent validity, and discriminant validity (*N* = 412).

Construct	*α*	*ω*	AVE	CR	√AVE	1	2	3	4
1. SEC	0.91	0.92	0.54	0.88	0.73	–			
2. Teaching efficacy	0.89	0.90	0.50	0.84	0.71	0.44	–		
3. POS	0.87	0.88	0.52	0.87	0.72	0.29	0.31	–	
4. Burnout	0.85	0.86	0.48	0.83	0.69	−0.40	−0.35	−0.26	–

*N* = 412. *α*, Cronbach’s alpha; *ω*, McDonald’s omega; AVE, average variance extracted; CR, composite reliability; √AVE, square root of AVE. Values on the diagonal (bold) represent √AVE; values below the diagonal represent inter-construct correlations. Discriminant validity is supported when √AVE for each construct exceeds its correlations with other constructs (Fornell and Larcker, 1981).

To further establish discriminant validity, three alternative models were compared with the hypothetical four-factor model (Table 4). A three-factor model (combining SEC and teaching efficacy), a two-factor model (combining all personal resources vs. burnout), and a one-factor model all demonstrated significantly worse fit. The *χ*^2^ difference tests confirmed that the four-factor model fit significantly better than all alternative models (Δ*χ*^2^ significant at *p* < 0.001), supporting the discriminant validity of the four constructs. Relative to the four-factor model, the three-factor, two-factor, and one-factor models produced ΔCFI values of −0.08, −0.16, and −0.32, and ΔRMSEA values of 0.021, 0.040, and 0.072, respectively, all exceeding conventional criteria for measurement equivalence (ΔCFI > 0.01; ΔRMSEA > 0.015), thereby further supporting the distinctiveness of the four constructs. The empirical association between SEC and teaching efficacy (*r* = 0.44), while substantial, is comparable to associations observed among related teacher constructs and is consistent with their theoretical status as distinct but related personal resources (see Discussion).

**Table 4:** Confirmatory factor analysis: model comparison (*N* = 412).

Model	*χ*^2^ (df)	CFI	TLI	RMSEA (90% CI)	SRMR	AIC	Δ*χ*^2^ (Δdf)	ΔCFI	ΔRMSEA
M1: Four-factor	373.35 (131)	0.94	0.92	0.067 [0.059, 0.075]	0.05	842	–	–	–
M2: Three-factor (SEC + TE)	555.35 (132)	0.86	0.83	0.088 [0.081, 0.095]	0.08	1,022	182.00 (1)^***^	−0.08	0.021
M3: Two-factor	761.12 (134)	0.78	0.74	0.107 [0.100, 0.114]	0.10	1,224	387.77 (3)^***^	−0.16	0.040
M4: One-factor	1222.04 (137)	0.62	0.56	0.139 [0.132, 0.146]	0.14	1,679	848.69 (6)^***^	−0.32	0.072

*N* = 412. SEC, social-emotional competence; TE, teaching efficacy. M2 combines SEC and TE into one factor; M3 combines all personal resources (SEC, TE, POS) into one factor vs. burnout; M4 combines all variables into a single factor. ^***^p < 0.001 for *χ*^2^ difference test comparing each alternative model to M1. Degrees of freedom for the *χ*^2^ difference tests correspond to the number of factor correlations constrained to unity in each alternative model (Δdf = 1, 3, and 6 for M2, M3, and M4, respectively); *χ*^2^/df ratios were 2.85, 4.21, 5.68, and 8.92 for M1 through M4. ΔCFI and ΔRMSEA are computed relative to the four-factor model (M1); values exceeding ΔCFI > 0.01 and ΔRMSEA > 0.015 indicate non-equivalence of the compared model.

### 3.4 Moderated mediation analysis

The moderated mediation model (PROCESS Model 7) was tested with SEC as the predictor, teaching efficacy as the mediator, burnout as the outcome, and POS as the moderator of the SEC-to-efficacy path. Gender, age, teaching experience, and school level were entered as covariates. The complete regression coefficients, including covariates, are presented in Table 5.

**Table 5:** Regression coefficients for the moderated mediation model (*N* = 412).

Predictor	*B*	SE	*t*	*p*	95% CI [LL, UL]
Teaching efficacy model (*R*^2^ = 0.405)					
SEC (X → Me, *a*)	0.39	0.06	6.50	<0.001	0.27, 0.51
POS (W)	0.15	0.05	3.00	0.003	0.05, 0.25
SEC × POS (interaction)	0.12	0.04	3.00	0.003	0.04, 0.20
Gender	−0.15	0.09	−1.67	0.096	−0.33, 0.03
Age	0.02	0.01	2.00	0.046	0.00, 0.04
Teaching experience	−0.01	0.01	−1.00	0.318	−0.03, 0.01
School level	−0.08	0.06	−1.33	0.184	−0.20, 0.04
Burnout model (*R*^2^ = 0.318)					
SEC (*c*′)	−0.28	0.05	−5.60	<0.001	−0.38, −0.18
Teaching efficacy (Me → Y, *b*)	−0.32	0.07	−4.57	<0.001	−0.46, −0.18
Gender	0.12	0.06	2.00	0.046	0.00, 0.24
Age	−0.01	0.01	−1.00	0.318	−0.03, 0.01
Teaching experience	0.003	0.01	0.30	0.766	−0.02, 0.03
School level	0.05	0.04	1.25	0.212	−0.03, 0.13
Total effect (c)	−0.40	0.05	−8.00	<0.001	−0.50, −0.30

B = unstandardized coefficient. SEC, social-emotional competence; POS, perceived organizational support; Me, mediator (teaching efficacy); Y, outcome (burnout). Gender coded 0 = male, 1 = female. School level coded 1 = primary, 2 = junior secondary, 3 = senior secondary. All continuous predictors were mean-centered. The interaction term contributed a significant incremental Δ*R*^2^ = 0.0133 (ΔF(1, 404) = 9.00, *p* = 0.003) beyond the main effects.

As shown in Table 5, the total effect of SEC on burnout was significant (*c* = −0.40, SE = 0.05, *t* = −8.00, *p* < 0.001). SEC was positively associated with teaching efficacy (*a* = 0.39, SE = 0.06, *t* = 6.50, *p* < 0.001), and teaching efficacy was negatively associated with burnout (*b* = −0.32, SE = 0.07, *t* = −4.57, *p* < 0.001). The direct effect of SEC on burnout remained significant after accounting for teaching efficacy (*c*′ = −0.28, SE = 0.05, *t* = −5.60, *p* < 0.001), indicating partial mediation. The full model accounted for 31.8% of the variance in burnout (*R*^2^ = 0.318, *F*(6, 405) = 31.48, *p* < 0.001) and 40.5% of the variance in teaching efficacy (*R*^2^ = 0.405, *F*(7, 404) = 39.30, *p* < 0.001). It should be noted that these *R*^2^ values reflect the explanatory power of the entire model—including SEC, teaching efficacy, POS, the interaction term, and all covariates—rather than the unique contribution of SEC alone.

Hypothesis 3 predicted that POS would moderate the positive SEC-teaching efficacy association. The interaction term (SEC × POS) was a significant predictor of teaching efficacy (*B* = 0.12, SE = 0.04, *t* = 3.00, *p* = 0.003). The interaction accounted for a significant incremental Δ*R*^2^ = 0.0133 (Δ*F*(1, 404) = 9.00, *p* = 0.003) beyond the main effects. Simple-slope analysis (Figure 2) revealed that the positive association between SEC and teaching efficacy was stronger among teachers with high POS (+1 SD; simple slope = 0.50, SE = 0.08, *t* = 6.25, *p* < 0.001) than among those with low POS (−1 SD; simple slope = 0.28, SE = 0.07, *t* = 4.00, *p* < 0.001). Thus, Hypothesis 3 was supported.

**Figure 2:**
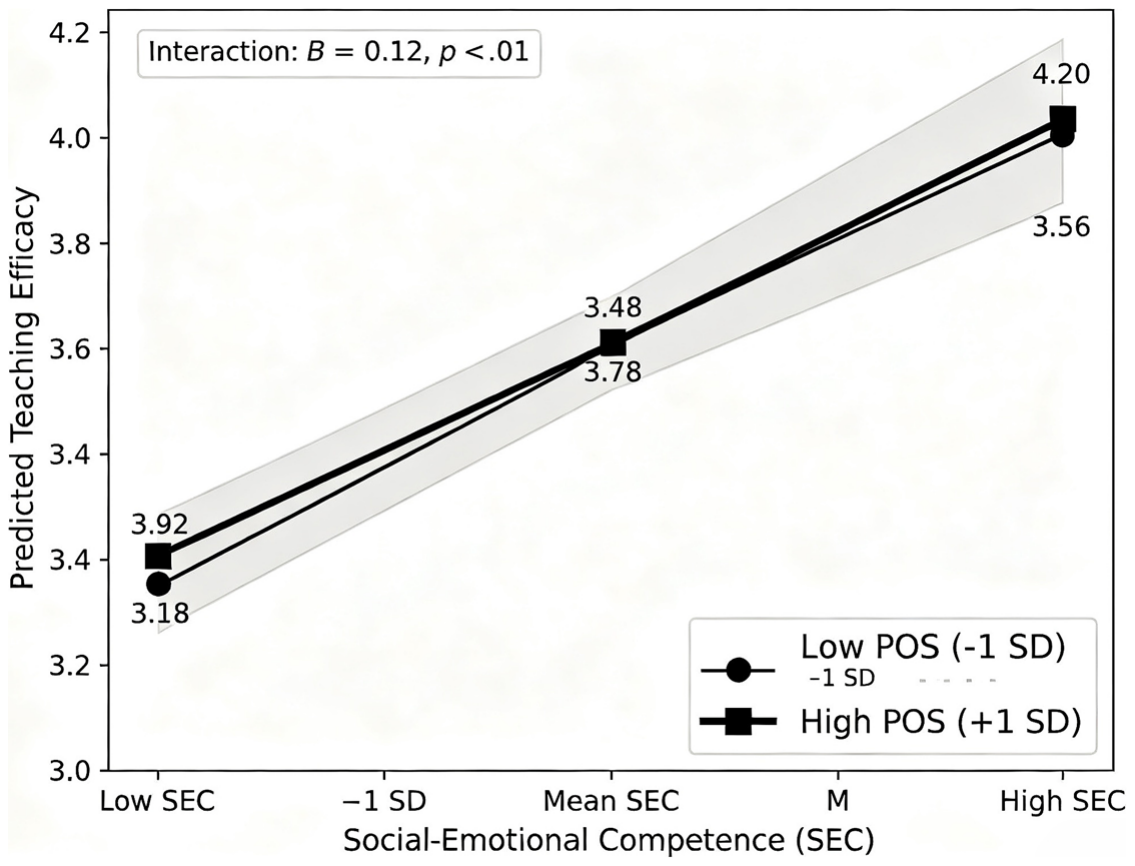
Simple slopes of teaching efficacy on SEC at high (+1 SD) and low (−1 SD) levels of perceived organizational support (POS). The positive association between SEC and teaching efficacy was stronger among teachers reporting high POS (solid line) than among those reporting low POS (dashed line). Shaded regions represent 95% confidence intervals.

The Johnson-Neyman analysis identified the transition point of POS at which the SEC-teaching efficacy simple slope shifted from non-significant to significant. The transition value was 3.42 (1.55 SD below the mean of POS, *M* = 4.85, SD = 0.92). For teachers with POS scores above 3.42, the SEC-teaching efficacy association was significantly positive. In the current sample, 93.9% of participants had POS scores above this threshold, indicating that the positive SEC-teaching efficacy association held for the vast majority of teachers in this study.

Hypothesis 4 concerned the conditional indirect effect of SEC on burnout through teaching efficacy at different levels of POS. As shown in Table 6, the indirect effect was significant at both high POS (IE = −0.160, 95% CI [−0.240, −0.088]) and low POS (IE = −0.090, 95% CI [−0.156, −0.034]). The index of moderated mediation was −0.038 (SE = 0.015, 95% CI [−0.068, −0.008]), indicating that the indirect effect was significantly stronger (i.e., more negative) at higher levels of POS. Because the confidence interval excluded zero, Hypothesis 4 was supported.

**Table 6:** Conditional indirect effects, index of moderated mediation, and effect ratios (*N* = 412).

POS level	IE	SE	Boot 95% CI [LL, UL]	IE/|c| (%)
Low (−1 SD)	−0.090	0.031	−0.156, −0.034	22.5
Mean	−0.125	0.030	−0.192, −0.065	31.2
High (+1 SD)	−0.160	0.039	−0.240, −0.088	40.0
Index of MM	−0.038	0.015	−0.068, −0.008	–

*N* = 412. SEC, social-emotional competence; POS, perceived organizational support; IE, indirect effect; MM, moderated mediation; IE/|c|, ratio of indirect effect to absolute total effect. Bootstrap sample size = 5,000. LL = lower limit; UL = upper limit of 95% bias-corrected confidence interval. J-N transition point = 3.42 (93.9% of sample above).

At the mean level of POS, the indirect effect through teaching efficacy was −0.125 (95% CI [−0.192, −0.065]), accounting for approximately 31.2% of the total effect (*c* = −0.40). At high POS, the indirect effect accounted for 40.0% of the total effect, whereas at low POS it accounted for only 22.5%. This indicates that organizational support is associated with a stronger proportion of the SEC-burnout association that operates through the teaching efficacy pathway.

In the sensitivity analyses, the alternative Model 14 specification, in which POS moderates the teaching efficacy-to-burnout path, yielded a non-significant interaction (*B* = −0.07, SE = 0.04, *t* = −1.75, *p* = 0.081) and a non-significant index of moderated mediation (−0.027, 95% CI [−0.059, 0.005]). Similarly, a specification in which POS moderates the direct SEC-burnout path produced no significant interaction (*B* = −0.04, SE = 0.03, *t* = −1.25, *p* = 0.213). These results indicate that the moderating role of POS was specific to the SEC-to-teaching efficacy pathway rather than reflecting a general tendency for POS to moderate all paths in the model.

## 4 Discussion

### 4.1 Principal findings

This study investigated the mechanisms by which English teachers’ social-emotional competence (SEC) relates to occupational burnout, focusing on the mediating role of teaching efficacy and the moderating role of perceived organizational support (POS). The findings provide converging evidence for a moderated mediation model: SEC was negatively associated with burnout (Hypothesis 1), teaching efficacy partially mediated this relationship (Hypothesis 2), POS moderated the SEC-to-efficacy pathway such that the association was stronger at higher POS (Hypothesis 3), and the indirect effect of SEC on burnout through teaching efficacy was conditional on POS, being stronger at high POS (Hypothesis 4).

### 4.2 Social-emotional competence, teaching efficacy, and burnout

The negative SEC-burnout association is consistent with the broaden-and-build theory (Fredrickson, 2001) and the Job Demands-Resources framework (Bakker and Demerouti, 2007). Teachers with higher SEC are better equipped to regulate their emotions, build supportive relationships, and make constructive decisions under pressure. These competencies serve as personal resources that may reduce vulnerability to the emotional exhaustion and depersonalization dimensions of burnout. Our finding is directionally consistent with meta-analytic evidence that emotional intelligence is negatively associated with burnout among foreign language teachers (Wang and Wang, 2022) and with evidence that teachers’ emotional intelligence is positively related to their self-efficacy and work engagement (Wang, 2022). The somewhat larger magnitude observed in the present study (*r* = −0.40) may partly reflect the multidimensional nature of the TSEC-S, which captures broader relational and decision-making competencies beyond emotional intelligence per se. However, this comparison should be interpreted with particular caution given that the TSEC-S, while demonstrating adequate psychometric properties in this study including pilot data on content validity, convergent validity, and test–retest reliability, is a recently developed measure whose construct validity requires further validation against established instruments in independent samples (see Limitations).

The partial mediation through teaching efficacy extends previous work linking teachers’ emotional competence to relationship quality and burnout (Poulou and Garner, 2024) and teaching efficacy to burnout (Kim and Burić, 2020; Hlado et al., 2025). From a theoretical standpoint, SEC may contribute to teaching efficacy because teachers who can perceive and manage classroom emotional dynamics are more likely to experience mastery in their instructional and classroom management efforts—the very experiences that build self-efficacy through enactive attainment (Bandura, 1997). Teaching efficacy, in turn, operates as a personal resource that may reduce vulnerability to burnout by promoting a sense of professional competence and reducing the appraisal of teaching demands as overwhelming. The indirect effect through teaching efficacy accounted for approximately 31% of the total SEC-burnout association, indicating a substantial but not predominant mediational pathway. The remaining 69% direct effect suggests that other mechanisms are also at play, potentially including direct emotional regulation effects, improved social support mobilization, and reduced rumination (Jennings and Greenberg, 2009; Poulou and Garner, 2024). It is worth emphasizing that SEC and teaching efficacy, although empirically associated (*r* = 0.44) and theoretically linked, are conceptually distinct constructs: SEC denotes a set of emotion-relevant, self-regulatory, and interpersonal competencies, whereas teaching efficacy denotes domain-specific beliefs about one’s capability to perform teaching tasks. The observed partial mediation is consistent with this conceptual distinction, indicating that SEC contributes to, but does not fully overlap with, teachers’ professional beliefs about their instructional effectiveness. At the same time, because both constructs were self-reported, shared self-perceptions may contribute to their observed association; the discriminant-validity evidence (√AVE exceeding the construct correlations) argues against, but cannot fully eliminate, this possibility.

### 4.3 The moderating role of perceived organizational support

A key finding is that POS moderated the SEC-to-teaching efficacy pathway. Among teachers who perceived high organizational support, the SEC-efficacy link was substantially stronger (simple slope = 0.50) than among those perceiving low support (simple slope = 0.28). The interaction accounted for a significant incremental Δ*R*^2^ = 0.0133 (*p* = 0.003) beyond the main effects. While this effect size is modest by conventional standards (Cohen, 1988), it is comparable to interaction effects typically observed in organizational field research (Aguinis et al., 2005) and carries meaningful theoretical implications. This finding is consistent with the JD-R model’s proposition that personal and job resources interact synergistically (Bakker and Demerouti, 2007), and with recent evidence that organizational and leadership conditions relate to teacher burnout through teachers’ social-emotional competence (Tian et al., 2022). When teachers work in an environment where they feel valued and supported, their social-emotional competencies may be more readily expressed in concrete teaching behaviors, recognized by students and administrators, and consolidated into a sense of professional mastery. Conversely, in low-support contexts, even highly socially-emotionally competent teachers may lack the autonomy, resources, and feedback necessary to translate their skills into efficacy beliefs. This pattern echoes the person-environment fit perspective: the realization of personal potential depends on the compatibility between individual capabilities and environmental affordances (Kurtessis et al., 2017).

The significant index of moderated mediation (−0.038, 95% CI [−0.068, −0.008]) confirms that the protective indirect effect of SEC on burnout—via teaching efficacy—varies as a function of POS. At high POS, the indirect effect was −0.160, whereas at low POS it was only −0.090, representing a 44% reduction in the mediational pathway under low-support conditions. The Johnson-Neyman analysis further revealed that the SEC-teaching efficacy association was significant for 93.9% of the sample, with only teachers reporting very low POS (below 3.42 on a 7-point scale) falling below the significance threshold. This finding has practical implications: organizational support does not merely buffer burnout directly but is associated with a stronger mechanism through which a key personal resource operates. As Ornaghi et al. (2023) noted, organizational climate influences the effectiveness of social-emotional processes on teacher outcomes. Our study extends this observation to naturally occurring SEC-burnout dynamics among English teachers.

### 4.4 The English teaching context in China

The specific relevance of this model for English teachers deserves elaboration. English language teaching in China involves high levels of emotional labor: teachers must maintain an enthusiastic, encouraging demeanor to lower students’ foreign language anxiety (Horwitz et al., 1986) while simultaneously managing the pressure of high-stakes examinations (Zheng, 2022; Yao et al., 2023). SEC is particularly important in this context because effective language instruction depends on the teacher’s ability to create a psychologically safe, emotionally engaging classroom environment. Our finding that the full model accounted for 40.5% of the variance in teaching efficacy and 31.8% of the variance in burnout underscores the centrality of social-emotional and organizational factors in the occupational wellbeing of English teachers. It should be emphasized that these *R*^2^ values reflect the combined contribution of SEC, POS, the interaction term, teaching efficacy, and all covariates, rather than the unique explanatory power of SEC alone. The cultural context of these demands also deserves emphasis. In Chinese schools, emotional expression is shaped by norms of restraint and harmony, teacher-student relationships carry distinctive hierarchical expectations that differ from Western settings (Fabris et al., 2023), and parental expectations regarding English attainment have intensified accountability pressures on teachers (Yao et al., 2023). Organizational support in Chinese schools, often expressed through leadership recognition, workload arrangements, and collective welfare provisions, may carry somewhat different meanings from Western conceptions of POS, and the present findings should be interpreted within this specific cultural frame (Hu et al., 2023; Mei and Alias, 2026). Testing these interpretations will require direct cross-cultural comparative work with matched samples of teachers.

### 4.5 Practical implications

From a practical standpoint, the findings suggest that interventions targeting both personal and organizational resources may be particularly promising for mitigating teacher burnout. First, teacher-training programs and professional development initiatives could systematically incorporate social-emotional learning components, which teachers themselves identify as central to their professional wellbeing (Denston et al., 2022) and which can be embedded within existing professional development structures (Masry-Herzallah, 2024; Li and Qian, 2025). Second, school administrators may benefit from cultivating organizational support structures—including supportive leadership, recognition of teacher contributions, professional autonomy, and access to resources—that enable teachers to translate their SEC into professional competence. The interactive pattern observed in this study is consistent with the proposition that personal resources and job resources operate jointly, but whether approaches that address both levels outperform approaches that target either level alone is an empirical question that intervention research, rather than the present cross-sectional design, is positioned to answer. We therefore offer these possibilities as directions to be tested rather than as conclusions.

### 4.6 Limitations and future directions

Several limitations should be acknowledged. First and foremost, the TSEC-S is a measure adapted from the CASEL framework for this study. Although pilot data provided preliminary evidence of reliability and validity—including acceptable internal consistency, convergent validity with the TEIQue-SF (*r* = 0.52), and test–retest reliability (*r* = 0.78)—the scale has not yet been validated in independent samples or against multiple established criterion measures. The absence of discriminant validity evidence distinguishing TSEC-S from related constructs such as emotional intelligence and personality traits limits the strength of conclusions that can be drawn specifically about SEC as a unique predictor. Future research should employ multitrait-multimethod designs and validate the TSEC-S against established teacher SEC measures. Consequently, the findings related to SEC should be considered preliminary pending further validation of the instrument. Second, the cross-sectional design precludes causal inferences. While the hypothesized directionality (SEC → efficacy → burnout) is grounded in theory, reverse causation and reciprocal effects are plausible. Longitudinal, experimental, and experience-sampling designs are needed to establish temporal precedence and causality; cross-sectional mediation estimates can be substantially biased when the underlying process unfolds over time (Maxwell and Cole, 2007; Loh and Ren, 2023). Third, all measures were self-reported, which may introduce common method bias and social desirability effects. Although both Harman’s single-factor test and the unmeasured latent method factor analysis did not indicate a serious threat, these procedures cannot rule out consistency motives, socially desirable responding, or shared negative affectivity as partial explanations of the observed covariation, and future studies should incorporate multi-source assessments, including student and supervisor ratings of SEC and burnout, objective workload and administrative records, informant reports, and social desirability markers. Fourth, the sample was drawn from three cities in Henan Province and may not be representative of English teachers across China. Fifth, the study focused on POS as a single contextual moderator; other job resources and job demands—particularly those central to the JD-R model such as workload, role conflict, and autonomy—may further shape the SEC-burnout nexus and should be examined in future research. Sixth, although CFA supported the latent variable structure of the measures, the moderated mediation analysis was conducted at the observed-variable level using PROCESS, which does not account for measurement error. Future research could employ latent variable moderated mediation (SEM-based) to obtain more precise effect estimates. Seventh, the data were collected from 24 schools, and although the ICC (0.043) indicated negligible clustering, we did not conduct multilevel modeling; future studies with larger cluster sizes should consider multilevel approaches. Eighth, we used a composite MBI-ES burnout score; while the dimensional intercorrelations supported this approach, dimension-specific analysis could reveal differential effects of SEC on emotional exhaustion, depersonalization, and personal accomplishment. Relatedly, the average variance extracted for the burnout composite (0.48) fell slightly below the 0.50 benchmark (Fornell and Larcker, 1981), indicating that the composite, although internally consistent (CR = 0.83), captured somewhat less than half of the variance in its indicators. Finally, the novelty of this study lies in its contextual specificity—examining the SEC-burnout nexus among English teachers—rather than in the moderated mediation model itself, which builds on established organizational behavior research. The incremental contribution of this study is the demonstration of these dynamics in a previously understudied population characterized by unique emotional labor demands. The full development and validation documentation for the TSEC-S is provided in the Supplementary material.

### 4.7 Conclusion

In conclusion, the present study demonstrates that English teachers’ social-emotional competence is associated with lower occupational burnout, both directly and indirectly through teaching efficacy. The moderating role of perceived organizational support indicates that organizational context shapes the extent to which personal resources translate into professional competence and, ultimately, into burnout protection. These findings contribute to a more nuanced understanding of the person-environment dynamics underlying teacher burnout and highlight the potential value of integrating personal and organizational approaches to supporting teacher wellbeing.

## Data Availability

The raw data supporting the conclusions of this article will be made available by the authors, without undue reservation.
