# Supplementary material for: English teachers’ social-emotional competence and occupational burnout: the mediating role of teaching efficacy and the moderating role of perceived organizational support
Source: Front Psychol. 2026 Sep 22;17:1972636. doi: 10.3389/fpsyg.2026.1972636 (PMC13638637; doi:10.3389/fpsyg.2026.1972636)
Supplement: Supplementary file 1 [file Data_Sheet_1.PDF]

Supplementary Material for:

English Teachers' Social-Emotional Competence and Occupational Burnout: The Mediating Role of Teaching Efficacy and the Moderating Role of Perceived Organizational Support

Manuscript ID: 1972636 (Frontiers in Psychology, Educational Psychology)

Supplementary Material 1. Development of the Teacher Social-Emotional Competence Scale (TSEC-S)

The Teacher Social-Emotional Competence Scale (TSEC-S) was developed in three stages. First, an initial pool of 30 items was generated on the basis of the five-competency framework of the Collaborative for Academic, Social, and Emotional Learning (CASEL) and the prosocial classroom model (Jennings and Greenberg, 2009), with all items adapted to the classroom teaching context and worded for a Chinese teacher population. Second, the item pool was reviewed by a panel of five independent experts (three specializing in social-emotional learning research and two in educational measurement), who rated the relevance of each item to its intended construct on a 4-point scale. The item-level content validity index (I-CVI) was computed as the proportion of experts rating the item 3 or 4; items below .80 were revised or deleted. Five items were removed at this stage (see Supplementary Material 2), yielding the final 25-item scale with a scale-level content validity index (S-CVI) of .89. Third, a pilot study with 50 English teachers (not included in the main sample) provided preliminary evidence of reliability and convergent validity, as summarized in Supplementary Material 3.

The TSEC-S was administered in Chinese. Table S1 reports the original Chinese items together with their English translations, which were prepared and back-translated by two bilingual researchers following a standard translation and back-translation procedure (Brislin, 1970). All items are rated on a 5-point Likert scale (1 = strongly disagree to 5 = strongly agree).

Table S1. Final 25 items of the TSEC-S (Chinese originals and English translations) with item-level content validity indices

Dimension	Item no.	Item content (Chinese original)	Item content (English translation)	I-CVI
Self-awareness	1	我能在课堂互动中准确识别自己的情绪。	I can identify my emotions accurately during classroom interactions.	1.00
Self-awareness	2	我能意识到自己	I am aware of how my mood affects	.89

		的情绪状态对教学的影响。	my teaching.	
Self-awareness	3	我能察觉自己何时开始对学生感到不耐烦。	I recognize when I am becoming frustrated with students.	.89
Self-awareness	4	我能注意到学生的情绪如何影响我自己的情绪状态。	I notice how students' emotions influence my own emotional state.	.80
Self-awareness	5	与同事交流时，我能清楚地表达自己的感受。	I can describe my feelings clearly when talking with colleagues.	.89
Self-management	6	课堂突发事件后，我能快速让自己平静下来。	I can calm myself quickly after a stressful classroom incident.	1.00
Self-management	7	面对学生的扰乱性行为时，我能控制自己的冲动反应。	I control my impulsive reactions when facing disruptive behavior.	.89
Self-management	8	在不同的教学情境中，我能恰当地调整自己的情绪表达。	I adjust my emotional expressions appropriately in different teaching situations.	.80
Self-management	9	我能应对工作压力，不让其累积成身心疲惫。	I manage work pressure without letting it accumulate into exhaustion.	.89
Self-management	10	我会留出时间反思自己在工作中的情绪体验。	I set aside time to reflect on my emotional experiences at work.	.89
Social awareness	11	即使学生不说，我也能察觉到他们的困扰。	I can tell when a student is distressed even without being told.	.89
Social awareness	12	我能理解来自不同家庭背景学生的情感需求。	I understand the emotional needs of students from diverse family backgrounds.	.80
Social awareness	13	我能察觉同事何	I recognize when colleagues are	.89

		时承受压力并需要支持。	under pressure and need support.	
Social awareness	14	学生在学习上遇到困难时，我能体谅他们的感受。	I appreciate students' feelings when they struggle with learning.	.89
Social awareness	15	学生出现挑战性行为时，我会站在他们的角度考虑问题。	I consider students' perspectives when they behave in challenging ways.	1.00
Relationship skills	16	我能与学生建立相互信任的关系。	I build trusting relationships with my students.	1.00
Relationship skills	17	我能以建设性、情绪平和的方式与家长沟通。	I communicate with parents in a constructive, emotionally balanced manner.	.89
Relationship skills	18	我能冷静而有效地化解与同事的冲突。	I resolve conflicts with colleagues calmly and effectively.	.89
Relationship skills	19	我能本着互助的精神与其他教师开展合作。	I cooperate with other teachers in a spirit of mutual support.	.80
Relationship skills	20	我能以尊重的方式表达对学校政策的不同意见。	I express disagreement with school policies respectfully.	.89
Responsible decision-making	21	我会评估自己的决策对学生身心发展造成的后果。	I evaluate the consequences of my decisions on students' wellbeing.	.89
Responsible decision-making	22	我会基于伦理考量作出课堂决策。	I make classroom decisions based on ethical considerations.	1.00
Responsible decision-making	23	我会为师生关系中的失误承担责任并主动修复。	I take responsibility for repairing mistakes in my relationships with students.	.80
Responsible decision-making	24	在教学上进行取舍权衡时，我会考虑学生的利	I weigh students' interests when making instructional trade-offs.	.89

		益。		
Responsible decision-making	25	我会考虑自己的职业选择对学生产生的长远影响。	I consider the long-term impact of my professional choices on my students.	.89

Supplementary Material 2. Items removed during expert review

The following five items were deleted from the initial 30-item pool on the basis of expert review and item-level content validity indices, together with the primary reason for removal.

Table S2. Deleted items and reasons for removal

Deleted item (Chinese original)	Deleted item (English translation)	I-CVI	Reason for removal
我了解学校的行政管理政策。	I understand administrative policies at my school.	.78	Judged peripheral to social-emotional competence
我能熟练而自信地使用教育技术。	I can use educational technology confidently.	.60	Content judged unrelated to the intended construct
我在任何时候都能保持积极的态度。	I maintain a positive attitude at all times.	.78	Absolute wording; high social desirability risk
我受到所有学生的喜爱。	I am liked by all of my students.	.60	Outcome statement rather than a competence; not behaviorally anchored
我能对学生完全隐藏自己的真实感受。	I can hide my true feelings from students completely.	.78	Inconsistent with the construct definition (suppression rather than competence)

Supplementary Material 3. Pilot study of the TSEC-S

A pilot study was conducted with 50 English teachers from the target population (not included in the main sample; 66.0% female; mean age 34.8 years, SD = 7.6; mean teaching experience 9.4 years, SD = 6.1) to evaluate the preliminary psychometric properties of the 25-item TSEC-S.

Table S3. Pilot study results (N = 50)

Property	Result
Internal consistency (total scale, Cronbach's alpha)	.89
Internal consistency (subscales, Cronbach's alpha)	.80 (responsible decision-making) to .86 (self-management)
Convergent validity: correlation with TEIQue-SF (Petrides, 2009)	$r = .52$, $p < .01$
Test-retest reliability (4-week interval, n = 42)	$r = .78$

Distribution	Skewness and kurtosis of the total score within ±1

Note. TEIQue-SF = Trait Emotional Intelligence Questionnaire Short Form. The pilot results were treated as preliminary evidence pending validation in the larger main sample (see Supplementary Material 4).

Supplementary Material 4. Five-factor CFA of the TSEC-S in the main sample (N = 412)

The five-factor measurement model of the TSEC-S (self-awareness, self-management, social awareness, relationship skills, responsible decision-making) was evaluated with confirmatory factor analysis using maximum likelihood estimation (AMOS 26.0) in the main sample.

Table S4. Fit indices of TSEC-S measurement models (N = 412)

Model	chi2/df	CFI	TLI	RMSEA (90% CI)	SRMR
Five-factor	2.41	.93	.91	.059 [.052, .066]	.05
Single-factor	6.74	.72	.68	.134 [.127, .141]	.11

Note. Standardized loadings on the five factors ranged from .53 to .81 (all $p < .001$). The five-factor model fit substantially better than the single-factor model (delta CFI = .21; delta RMSEA = .075).

Table S5. Factor correlations of the five-factor model (N = 412)

Factor	1	2	3	4	5
1. Self-awareness	—				
2. Self-management	.58	—			
3. Social awareness	.52	.55	—		
4. Relationship skills	.46	.44	.62	—	
5. Responsible decision-making	.41	.38	.49	.47	—

Note. All correlations significant at $p < .001$.

Discriminant considerations at the level of the four study constructs (average variance extracted, with √AVE exceeding the inter-construct correlations) are reported in Table 3 of the main text. Discriminant validity of the TSEC-S relative to related constructs, such as emotional intelligence and personality traits, remains to be established in independent validation samples.
